# An Apology

**Published:** 1874-12

**Authors:** 


An Apology is due our readers for the delay in the publication of the present number of our Journal. The increase in our circulation, which has trebled since September, must be accepted as our excuse.

